# Mujeres consumidoras de drogas en tratamiento ambulatorio: estudio cualitativo desde una perspectiva de género y de salud mental comunitaria

**DOI:** 10.18294/sc.2024.4648

**Published:** 2024-02-12

**Authors:** Noemí Águila-Morales, Rafael Clua-García

**Affiliations:** 1 Magíster en Salud Mental Comunitaria. Programa de Suport Individualitzat, Comunitat Terapèutica del Maresme, Malgrat de Mar, España. noemi.aguila.m@gmail.com Comunitat Terapèutica del Maresme Programa de Suport Individualitzat Comunitat Terapèutica del Maresme Malgrat de Mar España noemi.aguila.m@gmail.com; 2 Doctor en Antropología Social y Cultural. Profesor asociado, Facultat de Ciències de la Salut de Manresa, Universitat de Vic - Universitat Central de Catalunya, Manresa, España. rclua@umanresa.cat Universitat de Vic - Universitat Central de Catalunya Facultat de Ciències de la Salut de Manresa Universitat de Vic - Universitat Central de Catalunya Manresa España rclua@umanresa.cat

**Keywords:** Salud de la Mujer, Trastornos Relacionados con Sustancias, Servicios de Salud Comunitaria, Perspectiva de Género, Investigación Cualitativa, España, Women’s Health, Substance-Related Disorders, Community Health Services, Gender Perspective, Qualitative Research, Spain

## Abstract

Las mujeres consumidoras de drogas se enfrentan a grandes desafíos en el acceso y la adherencia al tratamiento ambulatorio. Sin embargo, esta cuestión ha sido poco estudiada. El objetivo del estudio es comprender las experiencias de un grupo de mujeres en tratamiento por consumo de drogas. Entre marzo y diciembre de 2021, se realizó un estudio cualitativo fenomenológico interpretativo mediante entrevistas semiestructuradas a 16 mujeres usuarias de un centro de atención a las drogodependencias de Cataluña (España). Los datos se procesaron mediante el análisis de contenido. Los resultados indican que las mujeres, pese a percibir un impacto positivo del tratamiento, viven realidades de vulnerabilidad relacionadas con la estigmatización, la falta de apoyo social y una baja cobertura de necesidades específicas con implicaciones en el seguimiento terapéutico. Los hallazgos enfatizan la necesidad de mejorar los recursos para una atención especializada y promover una red de apoyo social donde participen activamente las mujeres consumidoras de drogas.

## Abstract (en)

Women drug-users face significant challenges in accessing and adhering to outpatient treatment. However, this issue has been underexplored. The study aims to understand the experiences of a group of women undergoing drug use treatment. Between March and December 2021, a qualitative phenomenological interpretative study was carried out through semi-structured interviews with 16 women attending a drug-dependence care center in Catalonia, Spain. Data were processed through content analysis. The results indicate that, despite perceiving a positive impact of the treatment, women experience vulnerabilities related to stigmatization, lack of social support, and insufficient coverage of specific needs, impacting therapeutic follow-up. The findings emphasize the need to enhance resources for specialized care and promote a social support network where women drug-users actively participate.

## INTRODUCCIÓN

En el Estado español, como en otros países de Europa occidental, se estima que las mujeres representan entre el 25% y el 35% del total de personas consumidoras de drogas ilegales y entre el 45% y el 55% de drogas legales^1^^,^^2^. Sin embargo, las mujeres se encuentran subrepresentadas en los programas de tratamiento por abuso de sustancias, con una proporción de entre el 15% y el 25% del total de las admisiones^1^^,^^3^^,^^4^. Esto nos indica que las mujeres buscan con menor frecuencia el acceso al tratamiento y presentan bajas tasas de adherencia con respecto a los hombres. Diversos estudios resaltan que dichas barreras son consecuencia de las actitudes negativas hacia las mujeres consumidoras de drogas, la falta de apoyos sociales y una falla en una atención especializada desde los servicios sociosanitarios, que además generan mayor probabilidad de abandono del tratamiento y vulnerabilidad ante las recaídas^5^^,^^6^^,^^7^.

Aunque el consumo de drogas tiene menor incidencia en mujeres, estas presentan mayor vulnerabilidad y consecuencias sociales y de salud que los hombres. Las mujeres se exponen a mayor riesgo de las infecciones de transmisión sexual y sanguínea (VIH, hepatitis B y C, etc.)^8^^,^^9^, altas tasas de problemas de salud mental^10^, baja atención y acompañamiento durante el embarazo y el cuidado de las hijas e hijos^11^, sufrir diferentes tipos de violencia (abusos sexuales, violencia conyugal, etc.)^10^^,^^12^, así como a recibir estigmatización y criminalización^6^^,^^12^. Con ello, las mujeres experimentan altos niveles de culpabilidad y vergüenza de reconocer el consumo de sustancias y, a menudo, a normalizar la estigmatización como merecedora^6^^,^^11^. Además, las mujeres se enfrentan a la desigualdad en el acceso al empleo, sobrecarga en actividades de cuidados, opresión y vulneración sobre las decisiones y sobre sus propios cuerpos, entre otros factores, que modelan formas específicas de padecimientos subjetivos^13^.

En cuanto a la caracterización de los programas de los recursos asistenciales, en la mayoría de los casos no se incorpora la perspectiva de género y, por lo tanto, no se contemplan suficientemente las diferencias de género relacionadas con el consumo de drogas entre las mujeres^12^^,^^14^^,^^15^. En este sentido, el androcentrismo impregna la atención sanitaria, que conlleva a asumir una supuesta “neutralidad”, que invisibiliza las necesidades específicas al aplicarse las mismas intervenciones e interpretaciones en mujeres que en hombres y, por lo tanto, a que estas tengan una baja efectividad^16^.

En nuestro contexto geográfico, concretamente en Cataluña, se ha trazado un plan director que se fundamenta en un modelo comunitario que integra la atención a la salud mental y a las adicciones^17^. Específicamente, respecto a la intervención dirigida a personas consumidoras de drogas, se articula una red que proporciona atención sociosanitaria y comunitaria para todo tipo de sustancias en los centros de atención y seguimiento (CAS). En Cataluña se dispone de más de 60 CAS, que son centros ambulatorios y públicos, que proporcionan prevención, atención y soporte a personas adultas y sus familias. Los CAS cuentan con equipos multidisciplinares formados por profesionales de la salud y de atención social que prestan una atención biopsicosocial con programas de tratamiento y de reducción de daños a lo largo del proceso terapéutico de las personas usuarias. Desde los CAS se realizan derivaciones a otros servicios especializados de la red, como unidades hospitalarias, servicios sociales, entre otros^18^. En los últimos años se han realizado esfuerzos para una mayor cobertura de programas específicos y formación de las y los profesionales en cuanto a la intervención con perspectiva de género^19^, sin embargo, todavía no se ha estudiado suficientemente el impacto de este abordaje en la calidad de la atención de las mujeres consumidoras de drogas.

Las primeras aportaciones científicas sobre el consumo de drogas han proyectado una imagen de la mujer consumidora de drogas como una forma “desviada” de la feminidad “normal” y justificada, con frecuencia, como respuesta compensatoria a deficiencias físicas o mentales^20^; sin embargo, en los últimos veinte años, han aumentado los estudios que incluyen la perspectiva de las mujeres respecto al consumo de drogas. Entre estos, se destacan estudios que examinan la relación de las mujeres y la prescripción de psicofármacos^21^^,^^22^, las diferencias de género y el consumo de alcohol^23^^,^^24^^,^^25^^,^^26^, las trayectorias de mujeres consumidoras de opioides en tratamiento^27^, el consumo de cocaína en diferentes subpoblaciones de mujeres^28^^,^^29^, las experiencias de las mujeres consumidoras en prisión^30^ o sobre la estigmatización y las barreras de acceso a la atención sociosanitaria^12^^,^^31^. Dichos estudios evidencian una intervención deficitaria ausente de perspectiva de género. Además, son pocos los estudios cualitativos que hayan explorado las experiencias de las mujeres a lo largo del proceso terapéutico por uso de drogas. A nivel internacional, se conocen estudios desde las perspectivas de las mujeres acerca de las barreras de acceso al tratamiento de drogas^32^^,^^33^, los factores en la pérdida de adherencia y abandono del tratamiento^7^^,^^34^, el tratamiento de drogas y las dinámicas familiares^35^^,^^36^ y las necesidades en mujeres embarazadas y madres^37^^,^^38^.

Así pues, al explorar la bibliografía científica sobre este tema, detectamos que en la última década no se ha estudiado suficientemente acerca del consumo de drogas y el tratamiento dirigido a las mujeres en nuestro contexto geográfico. Además, se ha prestado menos atención a los desafíos que estas enfrentan a lo largo del proceso terapéutico en el contexto comunitario, razón que motivó la realización del presente estudio. Para lograr una mejor comprensión de las experiencias de estas mujeres, nuestra investigación integra un doble enfoque que vertebra la perspectiva de género con la de la salud mental comunitaria. 

Desde la perspectiva de género, atendemos al conjunto de roles, comportamientos, actividades y atributos socialmente construidos que una sociedad concreta considera apropiados para mujeres y hombres en toda su diversidad^20^^,^^39^. Específicamente, realizamos una aproximación a las realidades y contextos que generan desigualdades de género, ateniéndonos a que desde el orden patriarcal las mujeres han sido subordinadas e invisibilizadas respecto a los hombres. Aunque cada vez se está teniendo más presente la perspectiva de género en la intervención, generalmente, en los estudios sobre el uso de drogas, las mujeres no han sido suficientemente representadas, apartando esta dimensión como una cuestión periférica que niega sus experiencias^40^^,^^41^. En este sentido, exploramos las desigualdades y la discriminación que tienen implicación en el diseño y la aplicación de los programas en la atención comunitaria y, por ende, en la efectividad de las intervenciones^20^^,^^39^.

Siguiendo la estrategia del plan director de Cataluña, basado en la recuperación y la participación de la población como referentes en las intervenciones asistenciales^17^, dirigimos nuestro enfoque en salud mental comunitaria para ahondar en las instituciones y la comunidad, así como en los factores sociales y culturales que interceden en el bienestar de las personas consumidoras de drogas. Desde este planteamiento se cuestionan dicotomías de lo individual/social y biológico/psicológico, a partir de un paradigma científico basado en el cuidado y la ciudadanía, y para el cual se requiere una nueva estrategia y modelo teórico, organizativo y asistencial, dando paso a un modelo biopsicosocial^42^. En este sentido, nos centramos en las personas desde una mirada sistémica, poniendo énfasis en los derechos y la capacidad de agencia de estas, acogiendo la complejidad de lo colectivo y lo diverso^43^. Al respecto, dotamos de significado el sufrimiento individual a partir de la problemática y el malestar colectivo, más allá de la perspectiva organicista y biomédica desde la que suelen desatenderse otras demandas vinculadas a la salud mental como son la equidad, la participación ciudadana, así como el mundo narrativo de las personas afectadas, componentes que son clave para el diseño de estrategias de promoción de la salud^44^^,^^45^.

Con este doble enfoque se ha pretendido conocer con mayor profundidad las necesidades específicas de las mujeres consumidoras en tratamiento. Acorde a la bibliografía revisada, encontramos que existe la necesidad de tener en cuenta las características psicosociales y socioculturales de las mujeres y las necesidades específicas derivadas de éstas. Por este motivo, el presente estudio cualitativo se realizó con el objetivo de comprender las experiencias de un grupo de mujeres consumidoras de drogas a lo largo del tratamiento ambulatorio de las drogodependencias. Por lo tanto, a partir de la visión de las mujeres consumidoras de drogas, este estudio puso énfasis en repensar y reflexionar sobre elementos claves para una reorganización sanitaria y recursos comunitarios para una intervención más especializada y efectiva.

## METODOLOGÍA

Se realizó un estudio cualitativo de tipo fenomenológico interpretativo^46^^,^^47^. Para la recogida de datos se realizaron entrevistas semiestructuradas^48^ que permitieron establecer un diálogo de forma abierta y flexible para explorar los pensamientos, sentimientos y creencias de las participantes, así como para conceder espacio y significado a los discursos subjetivos de las mujeres como protagonistas de su tratamiento. De este modo, el diseño metodológico escogido nos condujo a la exploración y comprensión del mundo experiencial, emocional y subjetivo de las participantes.

El estudio se realizó entre marzo y diciembre de 2021 en el Centro de atención y seguimiento (CAS) a las drogodependencias de Calella (Barcelona), en el marco de una estancia formativa de la autora principal. Este centro se trata de uno de los más de 60 CAS que se disponen en Cataluña, ubicado a 50 km al norte de la ciudad de Barcelona. El CAS de Calella es el centro de referencia de un conjunto de municipios del norte de la comarca del Maresme con más 120 mil habitantes, que atienden alrededor de mil personas diferentes anualmente. En este se ofrece una atención especializada, sanitaria y social, a personas consumidoras de drogas, tanto a nivel individual como comunitario. 

El estudio fue diseñado por las dos autoras de este artículo, una psicóloga con cinco años de experiencia en el campo de la salud mental y un doctor en antropología y enfermero especialista en salud mental con una experiencia de 20 años. Durante los primeros meses de la investigación, la autora principal realizó un acercamiento con las posibles participantes del estudio y el entorno de su tratamiento. Junto con el segundo autor, que no participó directamente en la recogida de datos, compartió observaciones y reflexiones que fueron discutidas en reuniones para diseñar la estrategia de muestreo y el guion para la entrevista semiestructurada.

Se realizó un muestreo intencional^49^ con el fin de seleccionar y componer una muestra de mujeres usuarias del CAS de Calella que cumplieran con los criterios de selección. Buscábamos personas que se identificasen como mujeres, se encontraran en la etapa adulta y que tuviesen un seguimiento y adherencia regular al tratamiento. Fueron excluidas aquellas mujeres que presentaran condiciones físicas y cognitivas que impidieran seguir el curso de la entrevista, así como que presentaran una fuerte barrera idiomática. Las mujeres fueron propuestas a participar en el estudio por parte de la primera autora o bajo recomendación de las y los profesionales del CAS. Las participantes fueron contactadas de manera presencial, tras acudir a una visita con profesionales, o telefónicamente para concretar fecha y hora. El número de participantes se determinó siguiendo el principio de saturación^50^, mediante el cual el equipo investigador decidió que no se estaban obteniendo nuevas informaciones, lo que conllevó a la interrupción de la recogida de datos.

Las entrevistas semiestructuradas^48^ se realizaron entre julio y septiembre de 2021. Estas tuvieron una duración de alrededor una hora y se realizaron en castellano o catalán, dentro del CAS, en un espacio privado cedido por el servicio. El objetivo de la entrevista estaba dirigido a explorar las experiencias de las mujeres en relación con el tratamiento de drogas, realizando preguntas de forma abierta y flexible para obtener respuestas amplias desde el punto de vista de las participantes. El guion de la entrevista se estructuró en cinco bloques temáticos relacionados con: a) acceso y seguimiento al tratamiento; b) cobertura de las necesidades básicas; c) apoyos sociales; d) estigmatización; y e) propuestas de intervención. Mediante esta técnica fue posible una exploración personal, íntima y orientada a la subjetividad de las mujeres usuarias, dándole significado y espacio a su experiencia como válida y única. Además, mediante un pequeño cuestionario, se recogieron datos sociodemográficos, sobre el consumo de drogas y relacionados con el tratamiento.

Las entrevistas se trascribieron y fueron analizadas mediante el método de análisis de contenido^51^. Las trascripciones fueron asistidas para su codificación, utilizando el software cualitativo NVivo 12. En una primera fase, la primera autora realizó una lectura exhaustiva y en análisis de las transcripciones, utilizando códigos abiertos. Los resultados de este análisis preliminar fueron discutidos con el segundo autor con el fin de buscar patrones entre los códigos y obtener unos más precisos. Los códigos fueron ordenados en una tabla para facilitar la interpretación y la comparación sistemática de los datos recogidos. Con ello, en una segunda fase se propició un análisis más focalizado que aportó una mayor comprensión del problema de estudio. Se detectaron patrones recurrentes de significado (ideas, pensamientos y sentimientos) que se formalizaron en temas. Los resultados del análisis fueron discutidos en equipo con el objetivo de precisar las categorías y subcategorías enfocadas a dar explicación teórica y reflexiva de las experiencias de las mujeres consumidoras de drogas en tratamiento ambulatorio.

Todas las participantes fueron informadas de los objetivos y criterios éticos de la investigación. Las participantes firmaron un consentimiento informado siguiendo la Ley Orgánica 3/2018, de 5 diciembre, de protección de datos personales y garantía de derechos digitales, y el Reglamento de la UE 2016/679. El estudio fue realizado en el marco del trabajo final del Máster de Salud Mental Comunitaria de la Universitat de Barcelona, bajo la autorización de la dirección del CAS de Calella (Comunitat Terapèutica del Maresme - Serveis de Salut Mental). Este se realizó de acuerdo con los principios éticos de la Facultat de Psicologia de la Universitat de Barcelona, en consonancia con los principios éticos y el código de conducta de la American Psychological Association^52^ y la Declaración de Helsinki^53^.

## RESULTADOS

En el estudio participaron 16 mujeres con edades comprendidas entre 42 y 69 años con una media de 54,4 años. Catorce de las participantes eran de origen español. En relación con el nivel de estudios, siete tenían estudios secundarios, seis estudios primarios y tres universitarios. Respecto a la actividad laboral, seis se encontraban en situación de desempleo, cinco en incapacidad laboral, dos jubiladas y tres en actividad laboral. Cinco de las participantes percibían prestaciones económicas por incapacidad, tres prestaciones por desempleo, dos rentas mínimas de inserción, dos con prestación por jubilación y tres percibían sueldo laboral.

En relación con la familia, once vivían acompañadas de hijas e hijos, madres o pareja. Todas, excepto una, eran madres con una media de 1,5 hijos. Seis de las entrevistadas tenían hijas e hijos menores, entre las cuales dos habían sido intervenidas por el Equipo de Atención a la Infancia y Adolescencia (EAIA). Doce de las participantes verbalizaron haber recibido algún tipo de violencia (física, psicológica o sexual) producidas en el seno de una relación sentimental.

Respecto al tratamiento en el CAS, once de las participantes situaban el alcohol como la droga principal del motivo de consulta, entre las cuales seis asociaban este consumo con la cocaína. En conjunto, llevaban una media de nueve años en tratamiento por uso de drogas. Todas realizaban seguimiento psicológico y médico. Catorce estaban en tratamiento farmacológico por diagnósticos relacionados con trastornos del estado del ánimo. Siete participaban en grupos terapéuticos.

El análisis de los materiales recogidos mediante las entrevistas semiestructuradas permitió comprender las experiencias de las mujeres consumidoras de drogas en tratamiento ambulatorio. Presentamos los resultados en las tres categorías que surgieron durante el análisis de datos: 1) motivaciones para mantenerse en tratamiento; 2) barreras de acceso y de adherencia al tratamiento; y 3) propuestas para una atención especializada.

### Motivaciones para mantenerse en tratamiento

Las participantes del estudio presentaban una fuerte adherencia al tratamiento ofrecido desde el centro de atención y seguimiento (CAS) estudiado. Estas describían distintas motivaciones para mantener un vínculo con los servicios y profesionales del CAS, que se comprenden en tres subcategorías: resolver problemas sociales y de salud, sentirse apoyadas, y desarrollarse personal y socialmente.

Las motivaciones para acceder y mantenerse en tratamiento se relacionaban con la intención de mejorar su situación social y de salud. Las participantes, tras largos periodos de consumo de drogas y acumular experiencias negativas, encontraban el CAS como un espacio donde recibir una cobertura a sus necesidades. Específicamente, accedían al tratamiento con motivo de hacer frente a malestares que relacionaban con trastornos del estado del ánimo (ansiedad, depresión, estrés, etc.), más allá de los propios problemas de salud por un consumo intenso de drogas.

*No fue por el alcohol realmente, era porque tenía un problema de pareja. Todo fue culpa mía. Ella decidió que me fuera* [de casa] *y me hizo una orden judicial. Me dejó en la calle, así de claro.* (P10)

*Yo no vengo aquí por el tema de las drogas, vengo aquí porque me duele el alma y como me duele el alma, hago esta serie de cosas que hago.* (P15)

Las mujeres consumidoras de drogas se enfrentaban a dificultades sociales, económicas y laborales, siendo el cuidado de la familia uno de los motivos más comentados. En este sentido, las mujeres referían acudir a tratamiento para conseguir un ambiente familiar positivo y mejorar las relaciones intrafamiliares, sobre todo con las hijas y los hijos.

*Nos llamaron un día diciendo que preparara la ropa que se llevaban a la niña. Había drogas, malos tratos* [violencia recibida por parte de la expareja]. *Todo esto lo estoy haciendo por ella* [recuperar la tutela de su hija]. (P11)

*Realmente fue por ellos* [los hijos]*. No me lo pidieron, pero yo veía que estaban sufriendo.* (P4)

Otra de las claves de la adherencia al tratamiento era establecer una relación de ayuda con las y los profesionales del CAS. Las participantes referían tener buen vínculo con las y los profesionales de psicología, seguidos de los de enfermería, con las cuales percibían tener espacio de escucha, empatía, autoconocimiento, respeto y apoyo.

*Lo que más me ha ayudado es poder hablar, sin miedo. En casa no puedo hablar, ese es el problema.* (P1)

*En momentos que no he tenido a nadie me han hecho de enfermeras y psicólogas, me he sentido arropada y cuidada.* (P15)

Algunas de ellas también hacían mención a conceptos como “protección” y “seguridad” como necesidades vitales y que, en parte, son cubiertas en el CAS:

*A nivel social no he tenido apoyo. Me siento sola y perdida. Aquí en el CAS, me siento protegida…* (P14)

*Realmente a pesar de los trastornos y problemas que yo tengo, que no son pocos ni soy una persona fácil, siempre me he sentido protegida y apoyada. Es el motivo por el que yo vengo, porque necesito protección.* (P15)

Respecto a las intervenciones grupales, buena parte de las participantes expresaban haber encontrado un espacio donde tener soporte complementario al seguimiento individualizado. En este percibían apoyos con otras usuarias del CAS, sentirse parte de “algo” (pertenecer), y la oportunidad de compartir sus experiencias, generando así una red de apoyo entre iguales.

*No tengo amigos, no me relaciono con nadie. Me siento apoyada aquí y por Alcohólicos Anónimos. Tengo dos muletas muy importantes. Yo no me sentía parte de nada. Sentir que pertenezco a algo, que no soy un zombi.* (P6)

*Sí, volvería a participar en grupos. Es muy interesante encontrar mujeres que estamos todas más o menos cortadas por el mismo patrón. Compartir, aún con diferentes edades, diferentes motivos, pero para mí es muy enriquecedor.* (P4)

Finalmente, las motivaciones de las mujeres para vincularse al tratamiento se relacionaban con la adquisición de habilidades para desarrollarse social y personalmente. En general, las participantes hacían alusión a estabilizarse y conseguir una abstinencia absoluta del consumo de sustancias, con el objetivo de mejorar la calidad de vida y las relaciones sociales.

*Dedicarme a otras personas, conseguir entrada de dinero, pues he tenido que pedir dinero hasta para pagar el tren. Ser libre.* (P6)

*Estar con mi niña* [recuperar la custodia de la hija]*, aunque entienda que no pueda ser todos los días. Tener mi piso y un trabajito.* (P11)

En síntesis, sus objetivos y metas estaban estrechamente relacionadas con derechos básicos en nuestra sociedad y con necesidades básicas del devenir humano: seguridad, amor, pertenencia, y autorrealización. Desde aquí, tensiones vitales y malestares emocionales vinculados al consumo podían ser abordados de forma integral, social y comunitariamente durante el tratamiento, apaciguando así una parte importante del sufrimiento expresado por las mujeres usuarias.

### Barreras de acceso y de adherencia al tratamiento

Pese a que las mujeres mostraron una fuerte adherencia al tratamiento, estas lo iniciaban después de largas trayectorias de sufrimiento y de consumo intenso de drogas. Las mujeres expresaron distintas barreras de acceso que se comprenden en tres subcategorías: percibir estigmatización, sentir falta de apoyos y no recibir una atención ajustada a sus necesidades. 

Las participantes identificaban importantes dificultades para pedir ayuda que relacionaban estrechamente con la estigmatización. Estas expresaban haber recibido un trato diferenciado, sintiéndose juzgadas más negativamente que los hombres. Comentaban haberse sentido “avergonzadas” y a mantener un excesivo control para no dejarse “ver”. Además, sentían no recibir una atención empática al pedir ayuda en servicios de atención primaria y experimentar “malas experiencias”, percibiendo respuestas limitantes y obstaculizadoras.

*Al principio iba y decía que tenía un problema con el alcohol y me decían que no, que mis dolores eran emocionales, hasta que me vieron en urgencias. Ya tengo siete operaciones en mis manos. En el ambulatorio en este aspecto nunca me sentí escuchada, en ningún sentido.* (P6)

*He sentido juicio por consumir, soy una persona muy marcada. Incluso aunque dejara el consumo y yo me convirtiese en monja, estaría igual de señalada.* (P15)

Del mismo modo, algunas participantes comentaban no haber asistido a visitas o dejar pasar alternativas terapéuticas, como participar en grupos terapéuticos, por miedo a ser “descubiertas” como consumidoras en el ámbito laboral y/o familiar.

*Por eso te decía que no quise ir nunca a grupos de gente que hacía terapia, para no darme a conocer como consumidora.* (P7)

*Yo soy una persona que cuida mucho mi fachada, tengo una imagen, en mi trabajo, en mi vecindario. No he podido venir a visitas. He dejado pasar analíticas por no pedir permiso en el trabajo.* (P14)

Otra de las dificultades era la falta de apoyo a lo largo del seguimiento ambulatorio. La mayoría de las mujeres no se sentían apoyadas por su entorno cercano (familia, pareja, amistades…). Otras, aunque eran acompañadas durante el tratamiento, sintiendo la “presencia” y disponibilidad de personas de su entorno, percibían igualmente una falta de comprensión.

*La gente me aprecia, pero no saben que yo tengo un problema. No me siento apoyada. Me acompañan donde haga falta, pero no me acaban de entender.* (P1)

*Antes estaba fatal y muchas citas me las saltaba. Tenía una vida súper descontrolada. En ese momento me hubiera podido ayudar la persona que estaba conmigo, pero lo que me hizo fue desequilibrarme más.* (P9)

Respecto al seguimiento, las participantes percibían la necesidad de tener una mayor continuidad en las sesiones de tratamiento, sobre todo en momentos de crisis. Expresaban la necesidad de “sostén” en situaciones que no tenían dónde ni a quién acudir por una insuficiente red social. Las mujeres sentían la falta de asistencia como desencadenante a tener recaídas, con repercusiones en el tratamiento.

*Para mí lo ideal es cuando me siento mal, estoy sola en casa, entonces llamaría. No tener que esperar al día que me digan porque a lo mejor ese día no me ha pasado nada.* (P10)

*Cuando necesito ayuda, no llega en ese momento. Me desespero y caigo en el consumo. Me desconecto de mi tratamiento. Necesito más, el CAS se me queda corto.* (P14)

Las participantes también percibían una falta de individualización en los tratamientos, que ocasionaban una falta de continuidad en el seguimiento. Este aspecto generaba un sentimiento de desesperanza y la creencia de que eran “difíciles de ayudar”. En esta línea, aunque los servicios del CAS eran valorados positivamente, algunas participantes lo consideraban insuficiente para atender sus necesidades específicas (sufrir diferentes tipos de violencia, pérdida de trabajo, problemas económicos, etc.).

*Te sientes diferente, te encasillan en una cosa que no es, ¿entiendes? Porque había muchos hombres, si hay cincuenta personas, cuarenta y seis son hombres, ¿Qué pinto yo?* (P10)

*No quiero ingresar de nuevo, me han echado de todas partes. El tratamiento tiene que ser más específico.* (P15)

Además, una parte significativa de las participantes acusaba de ineficaces los cambios de referentes profesionales a lo largo del seguimiento. Las mujeres expresaban sentir pereza, miedo y resistencia de volver a explicar toda su historia y evolución, además de tener que formalizar una nueva vinculación con un o una profesional que no conocieran. Del mismo modo, se cuestionaba el sexo del profesional, percibiendo sentirse mejor comprendidas por mujeres profesionales, con las que existía un fuerte vínculo, para tratar temas compartidos entre mujeres.

*Era volver a empezar, volver a contar tus mierdas, a removerlo todo. He pasado por muchos profesionales. Te sientes muy desamparada, no te apetece y te dan ganas de tirar la toalla.* (P15)

*Yo creo que, si eres mujer y te atiende una mujer, hay conceptos que son mucho más fáciles de entender que si eres un hombre. No tienes que dar tantas explicaciones.* (P8)

Por último, y no por ello menos importante, las mujeres sentían que, en parte, el no recibir una atención ajustada a sus necesidades repercutía en una medicalización de sus malestares. Las participantes cuestionaban la intervención de psiquiatría, percibiendo una atención “escasa” e ineficaz acompañada de una percepción negativa respecto a los efectos secundarios de la medicación.

*El único problema es que los psiquiatras te dan muchas pastillas y llegó un momento que mi cerebro no funcionaba bien, no podía hablar. Para mí, fue horroroso eso.* (P1)

*Voy al psiquiatra ¿para decirle qué? si ya estoy yendo a un psicólogo. He ido a uno que le decía no sé qué y me llenaba de pastillas.* (P4)

### Propuestas para una atención especializada

A lo largo del trabajo de campo, las mujeres se expresaron de forma prudente respecto a cómo se sentían y con la satisfacción del seguimiento desde el CAS. En general, se mostraban agradecidas por la atención recibida. Pero al preguntar acerca de posibles mejoras en la intervención, muchas sintieron ciertas dificultades para realizar propuestas o argumentar desacuerdos respecto al tratamiento. En este sentido, en muchas ocasiones delegaban la responsabilidad de las indicaciones terapéuticas a las y los profesionales o se mostraban incapaces para formular opciones para una cobertura de necesidades específicas. Sin embargo, las participantes contemplaron alternativas para una atención especializada, que se comprenden en dos subcategorías: intervención individual e intervención grupal.

En relación con la intervención individual, las participantes expresaban la necesidad de tener un seguimiento más intenso por parte de las y los profesionales terapeutas (psicólogas y psicólogos). En general, las participantes expresaban que eran atendidas una vez al mes como media, percibiendo que esta atención y apoyo era insuficiente, por lo que la mayoría sentía la necesidad de que las visitas fueran con mayor frecuencia y duración.

*Entiendo que hay mucha gente, pero me gustaría seguir la pauta que diga mi doctora. ¿Egoístamente? Una vez a la semana, mejor.* (P6)

*Cuando estoy baja de ánimo, me gustaría venir dos veces a la semana, pero mínimo necesito una vez por semana. Tengo que contar con gente que me traiga y me da rabia, pero me gustaría hacerlo así, si pudiera ser.* (P11)

En esta línea, las mujeres manifestaban la necesidad de un trabajo terapéutico más continuado para enfrentarse a situaciones de riesgo de recaída. La mayoría de las participantes demandaba que existiera la posibilidad de tener acceso a un servicio específico, vinculado a la “emergencia”, para evitar situaciones de crisis. De acuerdo con esta necesidad, se proponía como opción un soporte telefónico para tener mayores posibilidades de atención en situaciones que requieren apoyo emocional, muchas veces escaso en su entorno.

*Yo lo único que veo, que a veces me gustaría que hubiera un sistema de que cuando estás en casa y tienes muchas ganas de beber, pueda avisar o llamar.* (P4)

*Una atención, aunque fuera telefónica, no presencial, que en ese momento me ayude a esa ansiedad que… tener ese apoyo, ese auxilio, ¿no? Me ayudaría muchísimo.* (P14)

Tanto para las sesiones de seguimiento individualizado como en la intervención de prevención de recaídas, se manifestaba la importancia de reforzar el trabajo terapéutico para adquirir más herramientas para el manejo de contingencias que conducen a un consumo intenso. Algunas mujeres, con largas trayectorias de consumo y seguimiento, expresaban de manera explícita la posibilidad de tener un vínculo indefinido con el CAS, es decir, sin la opción de ser dadas de alta por parte del servicio:

*A veces pasas por un sitio y dices: “va, ahora me tomaría una cerveza”. Solo el pensamiento y las ganas se consideraba una recaída. Cuando escribes* [en el momento] *te das cuenta de los problemas. Seria genial tener esa ayuda inmediata.* (P11)

*Continuar en el CAS, hasta que me dejen. Me gustaría tener seguimiento. Te dan el alta y te viene todo.* (P1)

En relación con la terapia grupal, las mujeres manifestaban la necesidad de poder involucrarse más en espacios colectivos para un seguimiento terapéutico complementario. Concretamente, comentaban la opción de participar en grupos terapéuticos para trabajar necesidades y motivaciones compartidas por las mujeres, como son el trabajo, la familia u otros intereses.

*Necesidad de hablar de mí, de mi vida. Espacios de compartir cosas de la vida, pero no hablar del consumo. Pienso que de mujer a mujer puede como que te puedan entender más*. (P2)

*Necesito sentirme que no estoy sola. Se podría hacer un grupo de mujeres que tengan más o menos las mismas cosas, que se puedan conectar para hablar, que estés acompañada.* (P16)

También, las mujeres hacían referencia a la opción de la terapia familiar con el fin de resolver conflictos y buscar cambios de dinámicas que mejoren el curso del seguimiento terapéutico. Más específicamente, manifestaban la necesidad de trabajar estrategias enfocadas en mejorar los apoyos con las hijas y los hijos, que faciliten la disposición de más espacio para realizarse en otros ámbitos.

*Más bien para relacionarme bien con la familia, que ellos entiendan mi situación y yo la de ellos.* (P9)

Él [hijo] *pensaba que era como un vicio, que lo podía dejar cuando me diera la gana a mí, que esto lo hacía porque quería. Me he ido distanciando.* (P10)

En definitiva, las mujeres hacían propuestas para ampliar apoyos y espacios para compartir y vincularse, sintiéndose “parte de” y “útil para” (sentido vital).

*Que una persona que tenga una dependencia, esté sin un trabajo, esto es un abismo. Que esa persona esté muy ocupada.* (P2)

*Hacer algo que me sienta yo útil. Estar con gente, tener pareja.* (P16)

## DISCUSIÓN

Este estudio nos ha permitido comprender las experiencias de un grupo de mujeres consumidoras de drogas en tratamiento ambulatorio. A partir del relato de las participantes hemos entretejido un conocimiento sobre las motivaciones para acceder y vincularse a un seguimiento terapéutico y afrontar malestares sociales y de salud. Sin embargo, hemos detectado múltiples barreras y limitaciones que presentan las mujeres relacionadas con la estigmatización, la falta de apoyos y una atención poco especializada a lo largo de sus itinerarios terapéuticos. Además, hemos recogido algunas de las propuestas de las participantes que apuntan a una reorganización de los recursos para impulsar intervenciones basadas en las fortalezas y capacidades de las mujeres y en la promoción de una red de apoyo social y comunitaria entre estas. Estos aspectos pueden ser de utilidad para las instituciones sociales y de salud en la puesta en marcha de políticas más inclusivas y ajustadas a las necesidades de las mujeres consumidoras de drogas.

Nuestros hallazgos nos indican que las mujeres consumidoras de drogas viven realidades de vulnerabilidad vinculadas a contextos de bajo soporte económico, social y comunitario. En consonancia con los estudios Wilson *et al*.^54^ y Salter *et al*.^55^, realizados con mujeres consumidoras de alcohol en Australia, las participantes expresaban estar expuestas a situaciones de elevado estrés relacionadas con tener sobrecargas familiares, sufrir diferentes tipos de violencia y percibir rechazo y exigencias de su entorno. A todo ello, se les suma un sentimiento de soledad profunda que, en su conjunto, otorga un mayor significado al malestar, estrechamente relacionado con iniciar y mantener un consumo intenso de drogas, como estrategia de refugio y evasión de la propia realidad experimentada. En concreto, nuestras participantes hacían consumos de alcohol, cocaína y psicofármacos como sustancias de preferencia. Estos se daban habitualmente en la esfera privada, “en secreto” para algunas de ellas, con un excesivo y minucioso control para no ser descubiertas como consumidoras de drogas. Así pues, las mujeres de nuestro estudio referían enfrentarse a grandes retos para manejar el doble estigma por ser mujer y consumidora de drogas y cumplir con las expectativas sociales exigidas y percibidas, tal como subrayan los estudios de Lee y Boeri^6^ y Meyers *et al*.^56^. Tras largos procesos de consumo oculto de las drogas, que deviene insostenible, las mujeres se movilizaban para pedir ayuda en servicios específicos.

Las motivaciones que llevan a las mujeres a buscar y mantenerse en tratamiento ambulatorio se relacionan de manera significativa con la necesidad de resolver malestares sociales y de salud, que no atañen directamente con el consumo intenso de drogas. En este sentido, las mujeres percibían el seguimiento terapéutico como estrategia para resolver problemas psicosociales, que implicaban cuadros de estrés y ansiedad relacionados con el mantenimiento del bienestar en el seno familiar, y sobrellevar otras obligaciones sociales, en consonancia con los estudios realizados en Brasil por Ribeiro *et al*.^34^ y Despontin *et al*.^57^.Para las participantes, el CAS representaba un servicio especializado que daba respuesta a buena parte de sus necesidades, de modo que asociaban el vínculo con las y los profesionales como uno de los pilares de mayor apoyo en sus vidas. Al respecto, las mujeres encontraban en el CAS un soporte emocional, en ausencia de apoyo social y comunitario, que se condice con lo expresado en los trabajos de Boroumandfar *et al*.^7^ y Llort *et al*.^31^. En nuestro estudio, las participantes con vínculos fuertes y duraderos percibían sentirse escuchadas y comprendidas, mejorando la confianza en sí mismas y, a su vez, reivindicaban la voluntad de compartir con el “otro” (relacional) y el pertenecer a “algo” (social).

Sin embargo, las mujeres expresaban enfrentarse a diferentes tipos de barreras a lo largo de sus itinerarios terapéuticos. De acuerdo con las revisiones bibliográficas de Motyka *et al*.^5^ y Meyers *et al*.^56^, uno de los principales desafíos se relacionaba con la acumulación de experiencias de rechazo por parte de profesionales de centros de atención social y de salud. Las participantes expresaban haber recibido estigmatización, que conducía a retrasar o evitar el inicio de tratamiento, así como a sentirse avergonzadas y a extremar la ocultación de su condición en el ámbito laboral y familiar, aspectos recogidos también en estudios realizados en Barcelona^12^ y EEUU^32^. De acuerdo con esta situación, muchas de las mujeres entrevistadas accedían solas a realizar el tratamiento por la falta de comprensión por parte de su entorno más cercano (familia, amistades, etc.)^7^. En esta misma línea, también se identificaron limitaciones con la percepción de no sentir una atención especializada y de no disponer de espacios suficientes para expresar sus preocupaciones o dudas. Las participantes expresaban recibir una atención centrada en el diagnóstico por trastorno mental, que implicaba una medicalización de los sentimientos y malestares, que aumentaba la percepción de una baja eficacia de los psicofármacos prescritos, tal como señalan los trabajos de del Río Pedraza^58^ y Bacigalupe *et al*.^59^. En relación con las y los profesionales, la mitad de las participantes expusieron sentirse mejor atendidas para tratar temas íntimos y personales que podían ser compartidos de forma libre y espontánea con mujeres profesionales que con hombres. 

Las mujeres, y en general las personas consumidoras de drogas, suelen recibir una atención paternalista que no les concede espacio suficiente para tomar decisiones por sí mismas. De acuerdo con esto, las participantes mostraron dificultad para hacer propuestas relativas a mejorar el plan terapéutico. No obstante, las mujeres hacían demandaban mayor frecuencia y tiempo en las visitas para establecer un seguimiento más profundo y de confianza con la profesional. En relación con esta cuestión, particularmente consideraban importante disponer de más apoyos en momento de crisis (por ejemplo, a través de un recurso telefónico de emergencia) para aliviar malestares o resolver dudas en la cotidianidad. Además, las participantes demandaban involucrarse en terapia grupal, exclusiva para mujeres, para poder sentirse cómodas para expresar emociones y necesidades compartidas con otras integrantes, lo que se condice con las recomendaciones de los estudios de Pallarés y Martínez^29^ y McCrady *et al*.^60^. A su vez, proponían participar en terapia familiar para mejorar la comunicación con familiares, especialmente con las hijas e hijos, así como resolver problemas relacionales en el seno de la familia, tal como evidencian los estudios de Goldberg *et al*.^35^ y Assalin *et al*.^61^. En su conjunto, estas propuestas se presentaban con el objetivo de fortalecer las relaciones y los vínculos (apoyos y cuidados) y como espacios para la expresión emocional del malestar. 

De acuerdo con nuestros hallazgos, es necesario que los servicios que dan atención a mujeres consumidoras de drogas amplíen su foco de intervención hacia un abordaje interseccional a lo largo del tratamiento ambulatorio, tal como señalan De Miguel-Calvo^30^ y Camarotti y Kornblit^62^. En concreto, y en consonancia con los trabajos de Simonelli *et al*.^63^ y Grella^64^, es importante una actuación que cuente con la especificidad de las realidades de las mujeres, poniendo mayor énfasis en el abordaje de los diferentes tipos de violencia, la falta de apoyo y la estigmatización que sufren a lo largo del proceso de recuperación. Del mismo modo, cuestiones referidas por las participantes, como las sobrecargas y exigencias del entorno, deben ser acogidas y abordadas con intervenciones específicas por parte de los recursos y servicios comunitarios. Por ello, actividades en y con la comunidad, que tengan como objetivo la toma de consciencia y el abordaje del estigma en las mujeres, son imprescindibles para superar uno de los obstáculos principales que enfrentan para acceder al tratamiento. En este sentido, intervenir e implicar al entorno cercano de las mujeres durante el tratamiento desde una mirada sistémica, promover la sensibilización y la formación de las y los profesionales en la perspectiva de género, así como dar la posibilidad de elegir el sexo del profesional como otras cuestiones que dificulten su seguimiento, y apostar más por los espacios de psicoterapia en detrimento de la farmacología son algunas de las posibles alternativas a las limitaciones sujetas a reflexión. 

En definitiva, es importante reenfocar el abordaje asistencial de los servicios desde una mirada comunitaria, incluyendo en las intervenciones los grupos de ayuda mutua y de apoyo, la creación de entornos que den espacio a la expresión y al soporte emocional, así como movilizando los recursos colectivos de los barrios y los centros de salud hacia un trabajo en red y hacia una reorganización de la propia red asistencial^12^^,^^31^. Específicamente, es recomendable proporcionar mayores espacios de intervención exclusivos para mujeres, que enfaticen una atención integral y de seguridad física, emocional y social^12^^,^^63^. Desde la perspectiva de género y en coherencia con la salud mental comunitaria, a través de la cual se recupera la condición de persona y ciudadana, más allá de la “enfermedad”, es necesario propiciar la intervención desde un contexto cálido y amigable para las mujeres. Y esto debe incluir la participación y, por ende, la validación de la voz de las mujeres como importantes promotoras de salud en la comunidad^65^^,^^66^. 

Este estudio está sujeto a varias limitaciones relacionadas con las características de la muestra. Se realizó con una muestra pequeña de mujeres cuyo principal motivo de consulta era por consumo de alcohol y, en segundo lugar, por cocaína consumida vía nasal, no habiéndose recogido datos sobre mujeres con otros tipos de consumo (consumo de drogas por vía parenteral y pulmonar). Además, nuestra muestra se completó con mujeres adultas, no teniendo acceso a mujeres jóvenes. Probablemente, dichas limitaciones se deban a la dificultad de reclutar a estas subpoblaciones de mujeres que, generalmente, presentan barreras de acceso a servicios sociosanitarios^5^^,^^12^. Además, la participación de personas migrantes fue baja, no pudiendo recogerse las especificaciones relacionadas con esta dimensión, respecto al tratamiento ambulatorio. Respecto a los datos recogidos, durante las entrevistas se presentaron dificultades para tratar temas, como los relativos a la sexualidad y su relación con la adicción. Así pues, es necesario realizar más estudios para ampliar nuestros hallazgos y poder ser considerados en la revisión de las estrategias de atención a las mujeres consumidoras de drogas. Recomendamos en un futuro realizar estudios con subpoblaciones no representadas en nuestra muestra, así como a profundizar en dimensiones que no fueron suficientemente recogidas y que son de interés para las mujeres estudiadas. 

## CONCLUSIONES

Las mujeres consumidoras de drogas se enfrentan diariamente a situaciones de vulnerabilidad relacionadas con sufrir estrés y sobrecargas familiares, padecer diferentes tipos de violencia y percibir estigmatización y exigencias en contextos de bajo apoyo económico, social y comunitario. Estas acceden y se mantienen en tratamiento motivadas con la necesidad de paliar malestares sociales y de salud. A lo largo del seguimiento terapéutico, las mujeres encuentran un soporte emocional por parte de las y los profesionales, que facilita recuperar la confianza y sentir mayor autorrealización. Sin embargo, las mujeres deben enfrentarse a múltiples barreras de acceso y de adhesión al tratamiento relacionadas con la estigmatización, la falta de apoyos y percibir una insuficiente cobertura de sus necesidades específicas. 

En esta línea, es necesario mejorar los recursos para una atención especializada y promover una red de apoyo social donde participen activamente las mujeres consumidoras de drogas. Para ello, es necesario el diseño de estrategias desde una perspectiva de género, en las que se ponga en el centro de la atención las necesidades reales de las mujeres, para configurar un tratamiento que eluda el androcentrismo imperante en los servicios de atención social y de salud. En un futuro, es imprescindible visibilizar las motivaciones y propuestas expresadas por las mujeres consumidoras de drogas, de manera de promover el empoderamiento y las devuelva al lugar que les pertenece en su comunidad. Precisamente, las necesidades de las mujeres se vinculan directamente con el mundo afectivo, relacional y de los apoyos, tan importantes para una salud individual y colectiva, lo cual nos conduce inevitablemente a la obligación de redefinir lo “sanitario” desde una mirada comunitaria
